# Costs, benefits and redundant mechanisms of adaption to chronic low-dose stress in yeast

**DOI:** 10.1080/15384101.2016.1218104

**Published:** 2016-08-11

**Authors:** Marta Markiewicz-Potoczny, David Lydall

**Affiliations:** Institute for Cell and Molecular Biosciences, The Medical School, Newcastle University, Newcastle upon Tyne, UK

**Keywords:** Adaptation, fitness, low-dose, stress, yeast

## Abstract

All organisms live in changeable, stressful environments. It has been reported that exposure to low-dose stresses or poisons can improve fitness. However, examining the effects of chronic low-dose chemical exposure is challenging. To address this issue we used temperature sensitive mutations affecting the yeast cell division cycle to induce low-dose stress for 40 generation times, or more. We examined *cdc13-1* mutants, defective in telomere function, and *cdc15-2* mutants, defective in mitotic kinase activity. We found that each stress induced similar adaptive responses. Stress-exposed cells became resistant to higher levels of stress but less fit, in comparison with unstressed cells, in conditions of low stress. The costs and benefits of adaptation to chronic stress were reversible. In the *cdc13-1* context we tested the effects of Rad9, a central player in the response to telomere defects, Exo1, a nuclease that degrades defective telomeres, and Msn2 and Msn4, 2 transcription factors that contribute to the environmental stress response. We also observed, as expected, that Rad9 and Exo1 modulated the response of cells to stress. In addition we observed that adaptation to stress could still occur in these contexts, with associated costs and benefits. We conclude that functionally redundant cellular networks control the adaptive responses to low dose chronic stress. Our data suggests that if organisms adapt to low dose stress it is helpful if stress continues or increases but harmful should stress levels reduce.

## Introduction

All organisms, from single celled bacteria and yeasts to complex mammals, experience a variety of environmental conditions, many of which are stressful. Organisms have evolved to adapt to different stresses and to thrive in varying conditions.^1^ Experiments in bacteria, yeast, worms, fish and mammals have revealed that different types of stress each induce common responses, termed the environmental stress response (ESR) or the cellular stress response (CSR).^2-8^ Responses induced by the ESR/CSR affect oxidative stress responses, protein folding, protein degradation, carbohydrate and fatty acid metabolism, DNA damage responses and intracellular signaling.^3,9-11^ Organisms exposed to stress often become resistant to higher levels of the same stress and cross-resistant to other stresses.^8,12-15^ The evidence is that a complex network of interactions regulates general as well as more specific responses to stress.^16-18^

**Figure 1.**
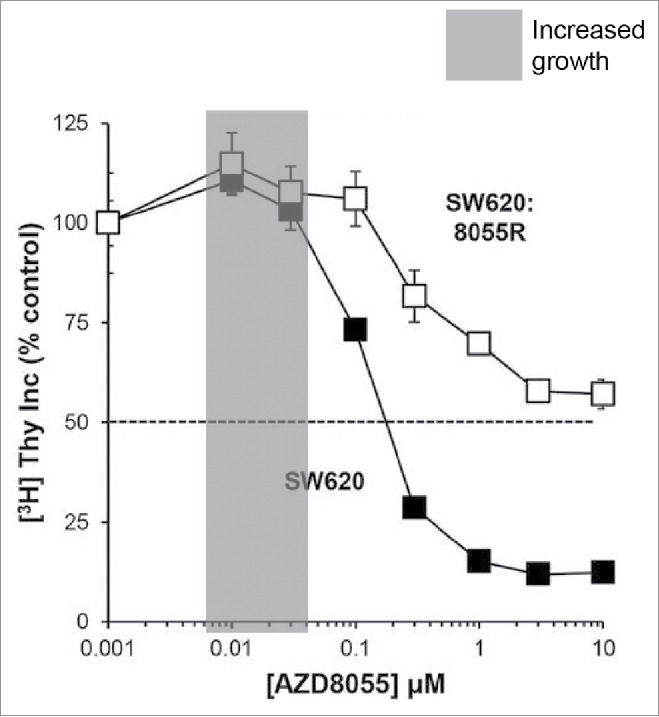
Hormetic response to mild stress in human cell lines. Human SW620 and SW620:8055R cell lines, the parental sensitive and a derived resistance line, were exposed to increasing concentrations of TOR kinase inhibitor, AZD8055, for 24 hours. Proliferation was assayed by [^3^H]thymidine incorporation. This figure is based on Figure 2 A of Cope *et al.*^26^. We added a gray box to highlight the area of increased fitness.

There is evidence that low-level stress can improve aspects of organismal fitness. In 1888 it was reported that yeast cells grew better when exposed to low-doses of poisons, such as arsenic trioxide, than in the absence of poison.^19^ This type of dose-response relationship, in which exposure to low-doses of a “poison” (e.g. environmental factor or chemical agent) improves some aspect of the fitness of an organism, is known today as hormesis. Hormesis has been reported in a variety of different systems from bacteria to yeast to vertebrates.^20-27^ A hormesis type response can often be observed in human cells growing in culture. Figure 1, adapted from Cope *et al*.,^26^ shows an example where growth of 2 human cancer cell lines, as measured by tritiated thymidine incorporation, is increased by low-doses of a Tor kinase inhibitor (Fig. 1).^26^ Numerous other studies with different biological end points, such as the effects of gamma radiation on mouse tumor formation or of DNA damage on worm longevity, support the idea that low-dose stresses can have a positive effect on organism fitness.^20,21, 25,27^

**Figure 2.**
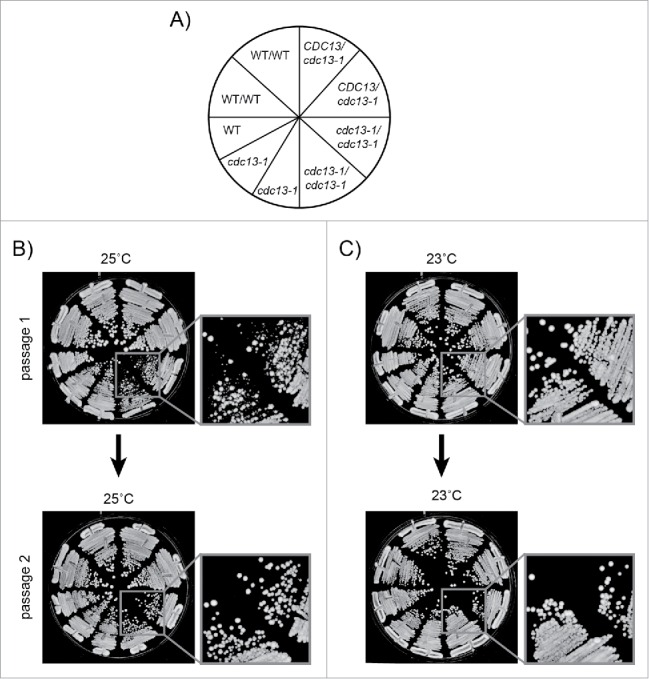
Passaging procedure. Freshly unfrozen strains were patched on YEPD agar plates and incubated at 23°C for 3 days, then passaged at 23°C or 25°C on YEPD agar plates as shown. 5–10 colonies from each genotype were pooled at each passage. Strains were: DDY81, 739, 738, 737, 736, 735, DLY1108, 1195, 3001. A) Genotypes of strains streaked on agar plates. B) Cells passaged for 2 passages at 25°C. C) Cells passaged for 2 passages at 23°C. DDY strains are diploids. DLY strains are haploids.

It is suggested that the biochemical mechanisms that explain hormesis are adaptive stress responses.^28-30^ Any amount of extrinsic stress will cause harm, for example by damaging DNA or other cellular components. However, it is thought up-regulation of maintenance and repair systems, in response to low-dose stress, protects not only from the extrinsic stress but also from intrinsic stresses that affect carcinogenesis, aging or other aspects of organismal fitness.^31-33^ If so, then perhaps it is mild induction of the ESR/CSR that explains the hormesis response.^14,34^ However, upregulation of maintenance and repair systems must come at some type of metabolic cost, otherwise organisms would never reduce maintenance and repair levels.^1^ These arguments suggest that hormesis is unlikely to occur very frequently.

When measuring the positive or negative effects of low-dose stress it is important to carefully consider what end point is measured. For example, mutations that extend lifespan in worms, and are considered positive by this criterion, also dramatically reduce the ability of worms to produce offspring in the laboratory environment and therefore have clear negative effects too.^35,36^ Moreover, the biochemical mechanisms underlying any positive responses to low dose stresses are poorly understood.^37-39^ The lack of good biochemical understanding of the response to chronic low-dose stresses or poisons stems, in part, because the effects induced are small and difficult to measure.^21,40^ Another difficulty, particularly for studies on the effects of chemicals, is that low-dose impurities, or chemical break down products, might confound interpretation. For example, at low concentrations the poison methotrexate improves viability of breast cancer cell lines.^41^ If methotrexate were contaminated with the chemically similar growth promoter, folate, then this might make interpretation of any positive effects of methotrexate difficult to distinguish from the effects of lower concentrations of the contaminant folate.

In this paper we carefully examined the responses of budding yeast, *Saccharomyces cerevisiae*, to chronic low-dose stress. Budding yeast is perhaps the best-studied model organism and much is known about its responses to stress.^42^ Although growth of yeast in the laboratory is artificial, for example with unlimited food supply or constant temperature, laboratory growth conditions mimic part of the natural yeast life cycle. It is thought that when yeast cells arrive on fruit, after being carried there by wasps, they divide rapidly from single cells.^43^ For this reason the rapid growth of yeast cultures in the laboratory is a biologically relevant measure of fitness.

To study the effects of chronic stress in yeast we used genetic mutations, rather than chemicals, to poison different aspects of the cell cycle. Temperature sensitive *cdc* (**c**ell **d**ivision **c**ycle) mutations affecting different aspects of cell cycle progression were used.^44,45^ We considered *cdc* mutations particularly attractive tools for this purpose because the dose of stress can be simply adjusted by controlling the culture temperature. The higher the temperature the more “poisoned” cell cycle events become. Furthermore, since each cell in the population carries the same mutation we can be sure that each cell in the environment is stressed to a similar extent. *cdc13-1* mutants are defective in telomere related functions. At high temperatures telomeres of *cdc13-1* cells induce a DNA damage response, akin to the response to DNA double strand breaks elsewhere in the genome.^46,47^ In this sense, the effect of *cdc13-1* mutation mimics that of genotoxic agents. Temperature sensitive *cdc15-2* mutants are defective in a kinase required for exit from mitosis and arrest cell division in late anaphase at high temperatures.^48^ Using these yeast genetic tools we asked: Does adaptation to chronic low-dose stress have a positive effect, a negative effect, neither, or both? We also addressed whether adaptation to stress is reversible and dependent on specific pathways.

## Results

### *cdc13-1* mutants adapt to chronic telomeric stress

To examine the response of yeast cells to chronic low-dose of telomere stress we used strains containing the *cdc13-1* allele, affecting the essential telomere capping protein Cdc13. *cdc13-1*, a point mutation, causes a P371S amino acid change and this induces temperature dependent Cdc13-1 protein degradation.^49,50^ In our lab *cdc13-1* strains are routinely cultured at 23°C (a permissive temperature). However, *cdc13-1* shows synthetic genetic interactions with mutations affecting the KU or MRX complexes at this temperature and thus we know that Cdc13-1 is not fully functional at 23°C.^51,52^ Therefore we also sometimes culture *cdc13-1* strains at 20°C, where Cdc13-1 is more functional. At higher temperatures *cdc13-1* mutants have dysfunctional telomeres, generate long telomeric 3′ ssDNA G-tails and activate Rad9-dependent cell cycle arrest.^46,53^ We have previously reported that *cdc13-1* mutants cultured at 30°C, a normal temperature for growing yeast, induce the ESR.^54^

It is known that many recessive, loss of function mutations, for example affecting DNA damage response (DDR) or nonsense mediated RNA decay (NMD) pathways, improve the fitness of *cdc13-1* strains grown at >26°C.^55,56^ Therefore we performed experiments in diploid cells to reduce the chance that recessive loss of function mutations affecting DDR, NMD or other genes were selected during our experiments. We passaged *cdc13-1* cells at 23°C, as usual, or at 25°C, a slightly higher temperature, to induce chronic low-dose telomere stress. 25°C slightly reduced the fitness (colony size) of *cdc13-1* mutants grown on agar plates, and therefore 25°C was considered the maximum permissive temperature^46^ (Supplementary Figure 1A). Interestingly, there was heterogeneity in colony size after the first passage at 25°C, but this disappeared by the second passage (Fig. 2B). This suggests that all cells in the population have adapted to the increased stress by the second passage. The heterogeneity in colony size was not observed when cells were passaged at 23°C (Fig. 2C).

**Figure 3.**
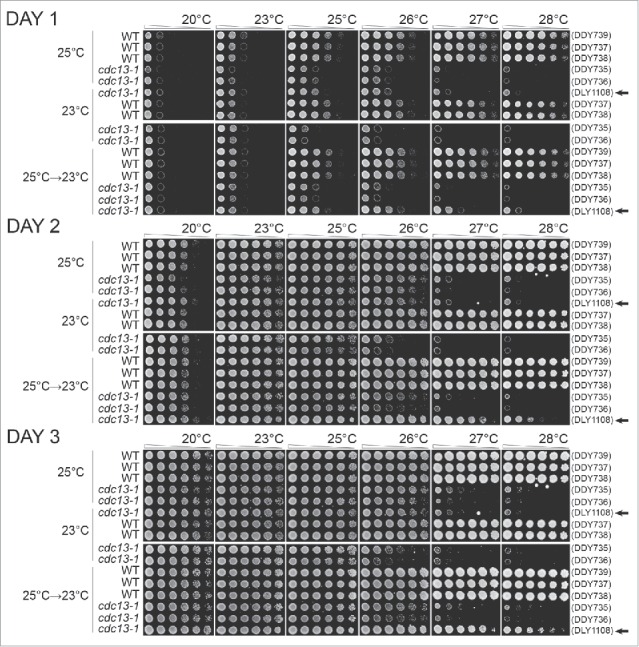
The effects of adaptation to low-level of telomere stress in *cdc13-1* mutants. Diploid strains with the genotypes indicated were grown on solid agar plates for 10 passages at 23°C, 10 passages at 25°C, or for 7 passages at 25°C followed by 3 passages at 23°C as indicated on the left of the Figure. Strains (DDY735, 736, 737, 738, 739 and DLY1108) were then inoculated into liquid and grown overnight at 23°C or 25°C. Fivefold dilution series of strains were set up in 96 well plates transferred to several independent solid YEPD agar plates using a pin tool. Individual plates were incubated at different temperatures, indicated across the top of the Figure, and photographs taken after 24, 48 and 72 hours of incubation. Arrows on right indicate haploid cultures.

Importantly, we never observed, at any temperature or time, that *cdc13-1* cells grew better than *CDC13* cells grown on the same plate (Fig. 3). By this criterion we see no evidence for a hormetic effect in this experimental system. In other words we see no evidence that exposure to chronic low-dose telomere stress improved fitness. However, we did observe that *cdc13-1* strains previously passaged at 25°C grew better at 26°C or 27°C than genetically identical strains passaged at 23°C (Fig. 3). We conclude that, as expected, adaptation to chronic mild telomere stress improves the fitness of strains exposed to even higher levels of telomere stress.

Interestingly, we observed that *cdc13-1* strains, previously passaged at 25°C, grew less well at 20°C than genetically identical strains previously passaged at 23°C. This phenomenon is telomere stress related, rather than temperature stress related, since equivalent *CDC13* cells, previously passaged at 23°C, were fully fit at 20°C. The simplest interpretation of these data is that the adaptation to telomere stress caused by growth at 25°C comes at a cost (trade off) affecting growth rate when telomere stress is reduced. Thus, in this context, adaptation to chronic telomere stress reduces fitness and is, in a sense, the opposite of hormetic.

Finally we tested whether the positive and negative effects of passaging *cdc13-1* cells at 25°C were reversible. Cells grown for 7 passages at 25°C were grown for a further 3 passages at 23°C before fitness was assessed. At 26°C such cells exhibited an intermediate growth phenotype, somewhere between the fitness of strains previously grown at 23°C or 25°C. Thus adaptation to telomere stress seems to be at least partially reversible. As predicted the haploid *cdc13-1* strain cultured at 25°C and then at 23°C did not reverse its ability to grow well at 26°C and above. Presumably this strain accumulated genetic mutations during growth at 25°C that suppressed *cdc13-1* defects. Interestingly, the cost of adaptation to growth at 25°C, seen at 20°C, seems to be fully reversed by growth for 3 passages at 23°C. Again, therefore, adaptation seems to be reversible and is, by this criterion, epigenetic rather than genetic.

### Adaptation to low-level of chronic telomeric stress is a robust response

Budding yeast responses to *cdc13-1* induced telomere defects are complex and have been well studied.^45,46, 54,56, 57^ We tested whether removal of Rad9, a central player in the response to *cdc13-1* induced telomere defects, affected adaptation. Rad9 was the first DNA damage checkpoint protein to be classified and plays at least 3 critically important roles in *cdc13-1* mutants. Rad9 is essential for cell cycle arrest of *cdc13-1* cells in metaphase, to inhibit ssDNA production near uncapped telomeres, and to maintain cell viability.^53,58-60^ Using temperature sensitive *cdc13-1 rad9Δ* mutants we performed analogous experiments to those performed on *cdc13-1* mutants. The maximum permissive temperature of *cdc13-1 rad9Δ* mutants is about 26°C, higher than *cdc13-1* mutants, presumably because low-levels of telomere damage induced at 26°C are sufficient to activate Rad9-dependent checkpoint pathways (inducing metaphase arrest), but insufficient to cause much cell death (as judged by colony size on plates) (Supplementary Figure **1A**).

**Figure 4.**
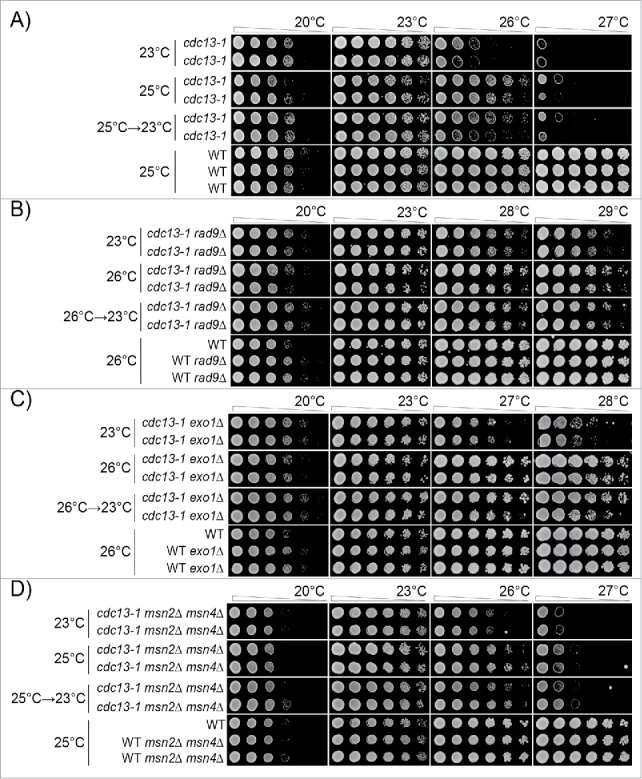
Adaptation to telomere stress in the absence of key stress response proteins. Diploid strains of different genotypes were analyzed as in Figure 3 except that strains were passaged twice at high temperature (25°C or 26°C), or once at high temperature followed by a single passage at low temperature, before being spotted onto agar plates. A) Strains as in Figure 3. Strains were DDY735, 736, 737, 738 and 739. B) Strains were DDY739; 868, 869, 860 and 861. C) Strains were DDY739, 992, 993, 994 and 995. D) Strains were DDY739, 974, 975, 972, 973. All strains shown in each subsection, at each temperature, were grown on single agar plates but images have been cut and pasted to make comparisons easier. Images were taken at 48 hours of incubation. Images taken after 24, 48 and 72 hours incubation are shown in Supplementary Figures: 2, 3 and 4, respectively.

Interestingly, we observed that *cdc13-1 rad9*Δ strains adapted after 2 passages at 26°C, similarly to *cdc13-1* strains at 25°C (Fig. 4A-B, Supplementary Figure 2). That is, *cdc13-1 rad9Δ* strains previously grown at 26°C grew better at higher temperatures, 28°C or 29°C, than genetically identical cells grown at 23°C (Fig. 4B). However, such cells exhibited poorer growth at lower temperatures, 20°C and 23°C. If *cdc13-1 rad9*Δ mutants were moved back from 26°C to 23°C, then the effects of adaptation were partially reversed. We conclude that *cdc13-1 rad9*Δ strains, like *cdc13-1* strains, can adapt to telomere damage and that the adaptation is reversible and comes with costs and benefits.

We also tested adaptation in the absence of Exo1, an exonuclease that degrades defective telomeres in *cdc13-1* strains.^61^ The maximum permissive temperature of *cdc13-1 exo1Δ* mutants is also about 26°C (Supplementary Figure 1B). Similarly to what was observed in *cdc13-1 rad9Δ* strains, we observed that *cdc13-1 exo1*Δ strains adapted after 2 passages at 26°C and grew better at 27°C and 28°C but exhibited poorer growth at lower temperatures, 20°C and 23°C (Fig. 4C, Supplementary Figure 3). When moved back from 26°C to 23°C, the effects of cells' adaptation were partially reversed. We conclude that *cdc13-1 exo1*Δ strains, like *cdc13-1* or *cdc13-1 rad9*Δ strains, can adapt to telomere damage and that the adaptation is reversible and comes with costs and benefits.

Finally we tested adaptation in the absence of Msn2/Msn4, 2 transcriptional activators that underlie the transcriptional response to numerous stresses.^2,62-65^ The maximum permissive temperature of *cdc13-1 msn2Δ msn4Δ* mutants is about 25°C (Supplementary Figure 1C). *cdc13-1 msn2*Δ *msn4*Δ strains adapted to 25°C after 2 passages, and grew better at 26°C, but exhibited poorer growth at lower temperatures, 20°C and 23°C (Fig. 4D, Supplementary Figure 4). As before, the effects of adaptation were partially reversed when cells were returned from 25°C to 23°C (Fig. 4D).

In summary Figure 4 clearly shows that loss of critically important components of the DNA damage or stress induction networks, active in *cdc13-1* strains, still permits *cdc13-1* strains to adapt to stress. Thus we conclude that adaptation to chronic telomere-induced stress involves a robust network of responses.

### Adaptation to mitotic kinase inhibition

**Figure 5.**
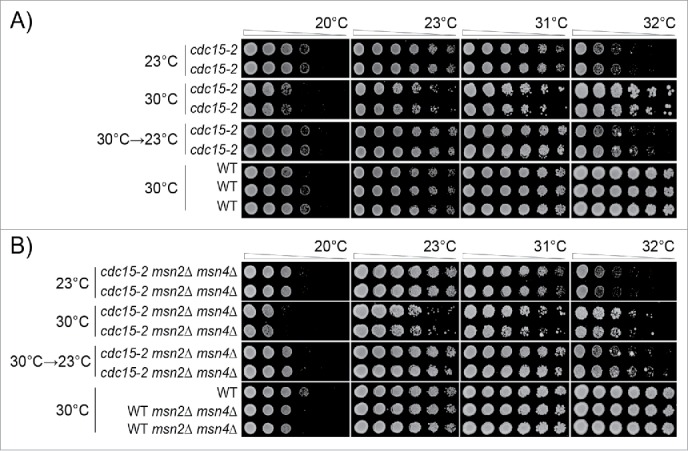
Adaptation to low-level kinase inhibition in *cdc15-2* mutants. Diploid strains of different genotypes were analyzed as in Figure 3. Strains were grown for 3 passages at 23°C or 30°C on agar plates, or for 1 passage at 30°C and 2 at 23°C. A) Strains were DDY739; 839, 840, 841, 842. B) Strains were DDY739; 1036, 1037 1038, 1039. All strains shown in each subsection, at each temperature, were grown on single agar plates. Images taken after 24, 48 and 72 hours incubation are shown in Supplementary Figures: 5 and 6, respectively.

Mammalian cells exposed to low-doses of Tor kinase inhibitor grow better than cells not exposed to the inhibitor (Fig. 1).^26^ We therefore extended our genetic studies to examine responses to low-level inhibition of kinase activity. We chose to examine mutations affecting the Cdc15 kinase, not a member of the Tor kinase family, but which is an essential component of the Mitotic Exit Network and is critical for the completion of mitosis.^66,67^ Using temperature sensitive *cdc15-2* mutants we performed analogous experiments to those performed in telomere defective *cdc13-1* mutants. Since the maximum permissive temperature of *cdc15-2* mutants is about 31°C, the temperature used to cause mild stress was 30°C (Supplementary Figure 1A, D). We determined that we could observe adaptation to growth at 30°C after as little as 3 passages (Fig. 5A, Supplementary Figure 5).

We could draw very similar conclusions about how cells adapt to *cdc15-2* induced kinase defects as we had drawn earlier for *cdc13-1* mutants. We never observed, at any temperature or time, that *cdc15-2* cells grew better than *CDC15* cells grown on the same plate (Fig. 5A, Supplementary Figure 5). By this criterion we see no evidence for a hormetic effect in *cdc15-2* strains. Analysis of *cdc15-2* mutants showed clear evidence for adaptation to kinase inhibition, a clear cost of adaption to kinase inhibition and strong evidence that adaptation was reversible (Fig. 5A). In fact, adaptation to *cdc15-2* stress seemed nearly fully reversible after 2 passages at 23°C (Fig. 5A).

We also examined the effects of Msn2/Msn4 in the context of Cdc15 defects. *cdc15-2 msn2*Δ *msn4*Δ mutants adapted after 2 passages to 30°C, to exhibit better growth at 31°C and 32°C but poorer growth at lower temperatures, 20°C and 23°C (Fig. 5B, Supplementary Figure 6). The effects of adaptation were partially reversed when moved from 30°C to 23°C (Fig. 5B).

## Discussion

**Figure 6.**
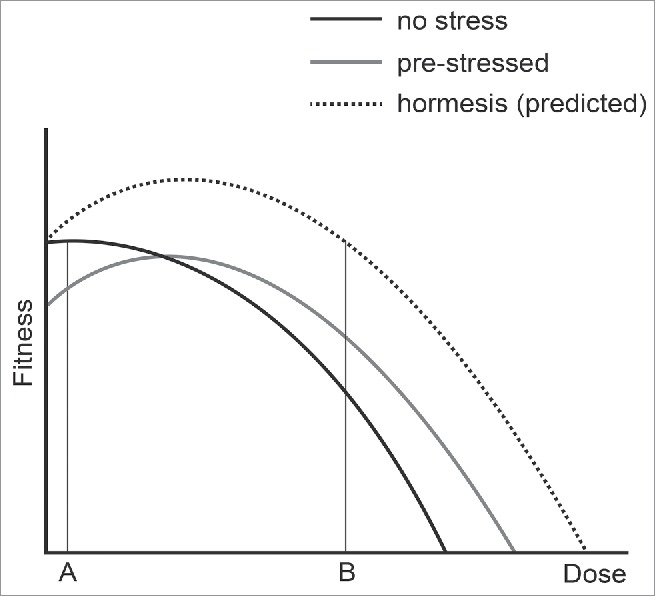
The effects of adaptation to mild stress. Three curves are used to represent the fitness of yeast cells that were previously not stressed (black line), pre-stressed (gray line) or show a hormetic response (dashed line). A and B indicate low and high doses of stress.

We analyzed yeast mutants defective in the cell division cycle to measure the costs and benefits of adapting to chronic low-level stress. We were particularly interested to determine if we could find evidence for hormesis, which has been defined as “a low-dose stimulation or beneficial effect and a high dose inhibitory or toxic effect,”^23^ in this simple model system. Figure 6 summarizes our observations and shows the positive and negative effects of adapting to different types of low-dose stress. We observe that previous exposure to low-dose stress improves the ability of yeast to survive higher doses of the same stress (Fig. 6, position B). However, the trade-off for adapting to low-dose stress is that cells are less fit, in comparison with unstressed cells, when grown in low stress conditions (Fig. 6, position A). We see no evidence for hormesis in this experimental system, that is that yeast cells exposed to low-dose stresses are fitter than cells not exposed to stress (Fig. 6, the predicted hormesis response curve, above the black dashed line). We cannot, of course, exclude that hormesis occurs in response to other types of stress, or could be observed if other biological end points were measured. For example, if we measured genetic stability rather than growth rate we might observe that cells with a slight induction of the DNA damage response were more stable than cells that had not induced the response.

What mechanisms might explain the different effects of the adaptive response to low-dose stress? *cdc13-1* and *cdc15-2* affect different aspects of cell cycle progression, but we imagine the types of adaptive response are somewhat similar. Defective telomeres in *cdc13-1* mutants induce a DNA damage checkpoint kinase dependent network, involving at least 5 kinases, Mec1, Tel1, Chk1, Rad53 and Dun1 to affect cell cycle arrest and DNA repair. Therefore effects on phosphorylation-dependent biochemical processes such as signaling, transcription and proteolysis must underlie the adaptive responses. Rad9, for example, is essential for cell cycle arrest of *cdc13-1* strains, for activating downstream kinase activities and for inhibiting the accumulation of single stranded DNA near telomeres.^47,53, 58,68-73^ Furthermore rearrangements in telomere DNA structure can also reduce cellular dependence on Cdc13.^74^

Since adaptation can occur in *cdc13-1 rad9Δ, cdc13-1 exo1Δ* and *cdc13-1 msn2Δ msn4Δ* strains, we conclude that much redundancy exists in the responses to telomere defects and that a strong network structure must underlie the adaptive responses. Consistent with this Berry *et al*.^16^ have shown acquired stress resistance is complex and context dependent. The fact that adaptation can occur in the absence of Rad9, which plays so many different and important roles in telomere defective strains, shows how robust (and presumably important) this adaptive response is. The complex network structure that underlies adaption may explain that numerous mechanisms have been proposed to account for hormetic mechanisms.^28-30, 37-39^ We have not examined the mechanisms that control adaptation to *cdc15-2* defects but we imagine that these mechanisms are equally as robust since Cdc15 is part of the mitotic exit network. We found that cells adapting to *cdc13-1* or *cdc15-2* induced stress incurred a detectable cost to fitness when the stress was reduced. Furthermore, cells seemed to reverse adaptation to *cdc15-2* stress more rapidly that they reversed adaptation to *cdc13-1* stress. This comparison suggests that the each type of stress can induce quite different adaptive mechanisms.

All organisms live in stressful, variable environments, encountering harmful physical and chemical stressors such as sunlight, radiation and oxygen. All of these poisons are unavoidably dangerous and cells have evolved powerful mechanisms to overcome most, but not all, of their harmful effects. It is likely that complex networks of biochemical interactions help cells tolerate and thrive in the context of these different stresses. Presumably, therefore, *cdc* mutants growing at slightly elevated temperatures up-regulated responses that counteract the negative cues caused by *cdc* defects. This up regulation, or adaptation, is positive because it prepares cells for exposure to even more stress. On the other hand adaptation is harmful when the stress is removed. In a sense *cdc* adapted cells have become “addicted” to stress, presumably because the energetic cost of maintaining a stress response when stress is no longer present reduces fitness.

In animals too there seems to be both a benefit and a cost of adapting to stress. Zebra finches exposed to heat stress in early life are more tolerant of heat later in life, but have shorter lifespans than unstressed birds if no further stress is received.^75^

*cdc13-1*, which essentially damages chromosomes, can be considered as a model for environmental stresses that cannot be avoided, such as radiation and oxygen exposure. We routinely grow *cdc13-1* cells at 23°C, although we know that telomeres of *cdc13-1* mutants at 23°C are imperfect, because synthetic lethal interactions are seen in combination with *yku70Δ, yku80Δ, mre11Δ, rad50Δ* and *xrs2Δ* mutations at this temperature.^51,52^ Presumably, therefore, the *cdc13-1* strains growing at 23°C have adapted to growth with defective telomeres. Along similar lines, most mammalian cells are grown in the laboratory in 20% oxygen, yet when cells have adapted to grow in lower oxygen concentrations and are switched back to 20% oxygen, then a strong stress response is observed.^76-78^ Similarly, all organisms are exposed to low-levels of radiation, but exposure can be increased environmentally. Interestingly, the most recent studies show that comparatively small radiation exposure in radiation workers leads to increased risk of death from leukemia.^79,80^ Our experiments measured growth rate, as a measure of fitness, but of course there are many other relevant measures of organism fitness. For example, it is likely that if we were to measure genetic stability of *cdc13-1* or *cdc15-2* cells grown at high temperature then stability would be reduced.

Experiments on mammalian cells in culture clearly show hormetic type responses to stress,^26^ so why do we not see evidence for hormesis in yeast? We favor the explanation that culture of transformed cancer cells in the laboratory does not reflect the carefully regulated cell growth that occurs in an organism. In tissue culture, perhaps, slightly inhibiting growth promoting signal transduction pathways (e.g., TOR) actually improves the fidelity of cell division and increases fitness. We suggest that the yeast experimental system is a better model for real organism growth. We cannot exclude, of course, that with a different experimental set up we might see hormesis in yeast, as was apparently seen in 1888.^81^

If organisms could be truly fitter after exposure to a toxin/poison/stress, then natural selection would surely select for organisms that generated or mimicked their own toxin/poison/stress. Environments change rapidly and it is not possible to predict what stresses are in store for the future. Our data suggests the idea that responses to chronic low-dose stress are not universally positive. The good news is that organisms can, if necessary, adapt to stress and this helps them survive increased stress. The bad news is that there is a price to be paid if the stress reduces. It has been suggested that positive responses to low-doses poisons will protect humans against cancer and/or aging.^21,27^ We caution against this view.

In summary our analyses of yeast cells exposed to chronic low-level telomere stress or mitotic kinase inhibition show clear evidence for adaption to stress. Adaptation improves cell fitness if cells are exposed to more of the same stress, but reduces fitness when stress levels are reduced. We found that adaptation to stress is reversible and that functionally redundant, robust networks of biochemical interactions must be responsible for the adaptive responses. Our results suggest that adaptation to low-level stress is favorable should stress remain or increase, but harmful when stress levels reduce.

## Materials and methods

### Yeast strains used in this study

All *S. cerevisiae* strains used in this study are in the W303 genetic background: *ade2-1 can1-100 trp1-1 leu2-3,112 his3-11,15 ura3 GAL+ psi+ ssd1-d2 RAD5+* and are shown in Supplementary Table 1.

### Media

Strains contain an *ade2-1* mutation and therefore the liquid or solid medium was supplemented with adenine (1L YEPD: 10g yeast extract, 20g bactopeptone, 50ml 40% dextrose, 15ml 0.5% adenine, 935ml H_2_0).

### Cell passage

Yeast strains were passaged on YEPD agar plates by streaking by toothpick at the indicated temperatures. Between 5 to 10 colonies from each genotype were pooled at each passage. We counted the cells in representative colonies and estimate that for each passage, for a single cell to form a colony represents approximately 21 cell generations.

### Spot test assays

Pooled colonies from cell passage plates were inoculated into 2ml liquid YEPD and grown on a wheel for 12-18 hours to reach saturation. Saturated liquid cultures were 5-fold serially diluted in sterile water in 96-well plates, and small aliquots were spotted onto YEPD plates with a sterile, metal pin-tool. Plates were incubated at the temperatures indicated and photographed at 24, 48 and 72 hours.

### Image processing

All agar plate images were processed using Adobe Photoshop CS6 software. In each Figure, all images were adjusted identically; there were slight differences across Figures. Images were adjusted as follows: in the Levels adjustment tool the low end of the intensity histogram was trimmed to between 100–130 and all other values left at default (1, 255). Using Photoshop's exposure tool, Exposure settings were adjusted to 0.3–0.8; Offset to −0.02–−0.05; and Gamma Correction to 1.2–1.5. Color was converted to grayscale after import into Adobe Illustrator.

## Supplementary Material

Supplemental Files

## Abbreviations

cdc: cell division cycle
ESR: environmental stress response
CSR: cellular stress response
DDR: DNA damage response
NMD: nonsense mediated RNA decay

## References

[1] Kirkwood TB. Understanding the odd science of aging. Cell 2005; 120:437-47; PMID:15734677; http://dx.doi.org/10.1016/j.cell.2005.01.027 15734677

[2] Gasch AP, Spellman PT, Kao CM, Carmel-Harel O, Eisen MB, Storz G, Botstein D, Brown PO. Genomic expression programs in the response of yeast cells to environmental changes. Mol Biol Cell 2000; 11:4241-57; PMID:11102521; http://dx.doi.org/10.1091/mbc.11.12.4241 11102521 PMC15070

[3] Kultz D. Molecular and evolutionary basis of the cellular stress response. Annu Rev Physiol 2005; 67:225-57; PMID:15709958; http://dx.doi.org/10.1146/annurev.physiol.67.040403.103635 15709958

[4] Lopez-Maury L, Marguerat S, Bahler J. Tuning gene expression to changing environments: from rapid responses to evolutionary adaptation. Nat Rev Genet 2008; 9:583-93; PMID:18591982; http://dx.doi.org/10.1038/nrg2398 18591982

[5] Schulte PM. What is environmental stress? Insights from fish living in a variable environment. J Exp Biol 2014; 217:23-34; PMID:24353201; http://dx.doi.org/10.1242/jeb.089722 24353201

[6] Young JW, Locke JC, Elowitz MB. Rate of environmental change determines stress response specificity. Proc Natl Acad Sci U S A 2013; 110:4140-5; PMID:23407164; http://dx.doi.org/10.1073/pnas.1213060110 23407164 PMC3593889

[7] de Nadal E, Ammerer G, Posas F. Controlling gene expression in response to stress. Nature reviews Genetics 2011; 12:833-45; PMID:22048664 22048664 10.1038/nrg3055

[8] Tauffenberger A, Parker JA. Heritable transmission of stress resistance by high dietary glucose in Caenorhabditis elegans. PLoS genetics 2014; 10:e1004346; PMID:24785260; http://dx.doi.org/10.1371/journal.pgen.1004346 24785260 PMC4006733

[9] Gasch AP The Environmental Stress Response: a common yeast response to environmental stresses In: Hohmann S, Mager P, eds. Yeast Stress Responses: Topics in Current Genetics. Springer-Verlag Heidelberg, 2002:11-70.

[10] Hahn A, Kilian J, Mohrholz A, Ladwig F, Peschke F, Dautel R, Harter K, Berendzen KW, Wanke D. Plant core environmental stress response genes are systemically coordinated during abiotic stresses. Int J Mol Sci 2013; 14:7617-41; PMID:23567274; http://dx.doi.org/10.3390/ijms14047617 23567274 PMC3645707

[11] Fulda S, Gorman AM, Hori O, Samali A. Cellular stress responses: cell survival and cell death. Int J Cell Biol 2010; 2010:214074; PMID:20182529 20182529 10.1155/2010/214074 PMC2825543

[12] Lewis JG, Learmonth RP, Watson K. Induction of heat, freezing and salt tolerance by heat and salt shock in Saccharomyces cerevisiae. Microbiology 1995; 141 ( Pt 3):687-94; PMID:7711907; http://dx.doi.org/10.1099/13500872-141-3-687 7711907

[13] Gasch AP. Comparative genomics of the environmental stress response in ascomycete fungi. Yeast 2007; 24:961-76; PMID:17605132; http://dx.doi.org/10.1002/yea.1512 17605132

[14] Milisav I, Poljsak B, Suput D. Adaptive response, evidence of cross-resistance and its potential clinical use. Int J Mol Sci 2012; 13:10771-806; PMID:23109822; http://dx.doi.org/10.3390/ijms130910771 23109822 PMC3472714

[15] Long Y, Yan J, Song G, Li X, Li X, Li Q, Cui Z. Transcriptional events co-regulated by hypoxia and cold stresses in Zebrafish larvae. BMC genomics 2015; 16:385; PMID:25975375; http://dx.doi.org/10.1186/s12864-015-1560-y 25975375 PMC4432979

[16] Berry DB, Guan Q, Hose J, Haroon S, Gebbia M, Heisler LE, Nislow C, Giaever G, Gasch AP. Multiple means to the same end: the genetic basis of acquired stress resistance in yeast. PLoS genetics 2011; 7:e1002353; PMID:22102822; http://dx.doi.org/10.1371/journal.pgen.1002353 22102822 PMC3213159

[17] Guan Q, Haroon S, Bravo DG, Will JL, Gasch AP. Cellular memory of acquired stress resistance in Saccharomyces cerevisiae. Genetics 2012; 192:495-505; PMID:22851651; http://dx.doi.org/10.1534/genetics.112.143016 22851651 PMC3454879

[18] Chasman D, Ho YH, Berry DB, Nemec CM, MacGilvray ME, Hose J, Merrill AE, Lee MV, Will JL, Coon JJ, et al. Pathway connectivity and signaling coordination in the yeast stress-activated signaling network. Mol Syst Biol 2014; 10:759; PMID:25411400; http://dx.doi.org/10.15252/msb.20145120 25411400 PMC4299600

[19] Schulz H Ueber Hefegifte. Pflügers Archiv - European Journal of Physiology 1888; 42:517-41; http://dx.doi.org/10.1007/BF01669373

[20] Calabrese EJ, Baldwin LA. Hormesis: the dose-response revolution. Ann Rev Pharmacol Toxicol 2003; 43:175-97; PMID:12195028; http://dx.doi.org/10.1146/annurev.pharmtox.43.100901.140223 12195028

[21] Calabrese EJ. Hormesis: a revolution in toxicology, risk assessment and medicine. EMBO Rep 2004; 5 Spec No:S37-40; PMID:15459733; http://dx.doi.org/10.1038/sj.embor.7400222 15459733 PMC1299203

[22] Calabrese EJ, Bachmann KA, Bailer AJ, Bolger PM, Borak J, Cai L, Cedergreen N, Cherian MG, Chiueh CC, Clarkson TW, et al. Biological stress response terminology: Integrating the concepts of adaptive response and preconditioning stress within a hormetic dose-response framework. Toxicol Appl Pharmacol 2007; 222:122-8; PMID:17459441; http://dx.doi.org/10.1016/j.taap.2007.02.015 17459441

[23] Mattson MP. Hormesis defined. Ageing Res Rev 2008; 7:1-7; PMID:18162444; http://dx.doi.org/10.1016/j.arr.2007.08.007 18162444 PMC2248601

[24] Costantini D, Metcalfe NB, Monaghan P. Ecological processes in a hormetic framework. Ecol Lett 2010; 13:1435-47; PMID:20849442; http://dx.doi.org/10.1111/j.1461-0248.2010.01531.x 20849442

[25] Ermolaeva MA, Segref A, Dakhovnik A, Ou HL, Schneider JI, Utermohlen O, Hoppe T, Schumacher B. DNA damage in germ cells induces an innate immune response that triggers systemic stress resistance. Nature 2013; 501:416-20; PMID:23975097; http://dx.doi.org/10.1038/nature12452 23975097 PMC4120807

[26] Cope CL, Gilley R, Balmanno K, Sale MJ, Howarth KD, Hampson M, Smith PD, Guichard SM, Cook SJ. Adaptation to mTOR kinase inhibitors by amplification of eIF4E to maintain cap-dependent translation. J Cell Sci 2014; 127:788-800; PMID:24363449; http://dx.doi.org/10.1242/jcs.137588 24363449

[27] Calabrese EJ, Shamoun DY, Hanekamp JC. Cancer risk assessment: Optimizing human health through linear dose-response models. Food Chem Toxicol 2015; 81:137-40; PMID:25916915; http://dx.doi.org/10.1016/j.fct.2015.04.023 25916915

[28] Stranahan AM, Mattson MP. Recruiting adaptive cellular stress responses for successful brain ageing. Nat Rev Neurosci 2012; 13:209-16; PMID:22251954 22251954 10.1038/nrn3151 PMC4084510

[29] Perry MC, Dufour CR, Eichner LJ, Tsang DW, Deblois G, Muller WJ, Giguere V. ERBB2 deficiency alters an E2F-1-dependent adaptive stress response and leads to cardiac dysfunction. Mol Cell Biol 2014; 34:4232-43; PMID:25246633; http://dx.doi.org/10.1128/MCB.00895-14 25246633 PMC4248744

[30] Singh F, Charles AL, Schlagowski AI, Bouitbir J, Bonifacio A, Piquard F, Krahenbuhl S, Geny B, Zoll J. Reductive stress impairs myoblasts mitochondrial function and triggers mitochondrial hormesis. Biochim Biophys Acta 2015; 1853:1574-85; PMID:25769432; http://dx.doi.org/10.1016/j.bbamcr.2015.03.006 25769432

[31] Haendeler J, Tischler V, Hoffmann J, Zeiher AM, Dimmeler S. Low doses of reactive oxygen species protect endothelial cells from apoptosis by increasing thioredoxin-1 expression. FEBS Lett 2004; 577:427-33; PMID:15556622; http://dx.doi.org/10.1016/j.febslet.2004.10.041 15556622

[32] Watson A, Mata J, Bahler J, Carr A, Humphrey T. Global gene expression responses of fission yeast to ionizing radiation. Mol Biol Cell 2004; 15:851-60; PMID:14668484; http://dx.doi.org/10.1091/mbc.E03-08-0569 14668484 PMC329398

[33] Christmann M, Kaina B. Transcriptional regulation of human DNA repair genes following genotoxic stress: trigger mechanisms, inducible responses and genotoxic adaptation. Nucleic Acids Res 2013; 41:8403-20; PMID:23892398; http://dx.doi.org/10.1093/nar/gkt635 23892398 PMC3794595

[34] Douglas H. Science, hormesis and regulation. Hum Exp Toxicol 2008; 27:603-7; PMID:19029255; http://dx.doi.org/10.1177/0960327108098493 19029255

[35] Walker DW, McColl G, Jenkins NL, Harris J, Lithgow GJ. Evolution of lifespan in C. elegans. Nature 2000; 405:296-7; PMID:10830948; http://dx.doi.org/10.1038/35012693 10830948

[36] Jenkins NL, McColl G, Lithgow GJ. Fitness cost of extended lifespan in Caenorhabditis elegans. Proc Biol Sci 2004; 271:2523-6; PMID:15590605; http://dx.doi.org/10.1098/rspb.2004.2897 15590605 PMC1440519

[37] Schumacher B. Transcription-blocking DNA damage in aging: a mechanism for hormesis. Bioessays 2009; 31:1347-56; PMID:19921662; http://dx.doi.org/10.1002/bies.200900107 19921662

[38] Vaiserman AM. Hormesis, adaptive epigenetic reorganization, and implications for human health and longevity. Dose Response 2010; 8:16-21; PMID:20221294; http://dx.doi.org/10.2203/dose-response.09-014.Vaiserman 20221294 PMC2836156

[39] Wiegant FA, de Poot SA, Boers-Trilles VE, Schreij AM. Hormesis and Cellular Quality Control: A Possible Explanation for the Molecular Mechanisms that Underlie the Benefits of Mild Stress. Dose Response 2012; 11:413-30; PMID:23983668; http://dx.doi.org/10.2203/dose-response.12-030.Wiegant 23983668 PMC3748852

[40] Peake JM, Markworth JF, Nosaka K, Raastad T, Wadley GD, Coffey VG. Modulating exercise-induced hormesis: Does less equal more? J Appl Physiol (1985) 2015; 119:172-89; PMID:25977451; http://dx.doi.org/10.1152/japplphysiol.01055.2014 25977451

[41] Kelly T, Owusu-Apenten RK Effect of methotrexate and tea polyphenols on the viability and oxidative stress in MDA-MB-231 breast cancer cells. J App Life Sci Int 2015; 2:152-9; http://dx.doi.org/10.9734/JALSI/2015/14142

[42] Botstein D, Fink GR. Yeast: an experimental organism for 21st Century biology. Genetics 2011; 189:695-704; PMID:22084421; http://dx.doi.org/10.1534/genetics.111.130765 22084421 PMC3213361

[43] Liti G. The fascinating and secret wild life of the budding yeast S. cerevisiae. Elife 2015; 4:1-9; PMID:25807086; http://dx.doi.org/10.7554/eLife.05835 25807086 PMC4373461

[44] Hartwell LH, Mortimer RK, Culotti J, Culotti M. Genetic Control of the Cell Division Cycle in Yeast: V. Genetic Analysis of cdc Mutants. Genetics 1973; 74:267-86; PMID:17248617 17248617 10.1093/genetics/74.2.267 PMC1212945

[45] Weinert TA, Hartwell LH. Cell cycle arrest of cdc mutants and specificity of the RAD9 checkpoint. Genetics 1993; 134:63-80; PMID:8514150 8514150 10.1093/genetics/134.1.63 PMC1205445

[46] Garvik B, Carson M, Hartwell L. Single-stranded DNA arising at telomeres in cdc13 mutants may constitute a specific signal for the RAD9 checkpoint. Mol Cell Biol 1995; 15:6128-38; PMID:7565765; http://dx.doi.org/10.1128/MCB.15.11.6128 7565765 PMC230864

[47] Lydall D. Taming the tiger by the tail: modulation of DNA damage responses by telomeres. EMBO J 2009; 28:2174-87; PMID:19629039; http://dx.doi.org/10.1038/emboj.2009.176 19629039 PMC2722249

[48] Schweitzer B, Philippsen P. CDC15, an essential cell cycle gene in Saccharomyces cerevisiae, encodes a protein kinase domain. Yeast 1991; 7:265-73; PMID:1882551; http://dx.doi.org/10.1002/yea.320070308 1882551

[49] Hughes TR, Weilbaecher RG, Walterscheid M, Lundblad V. Identification of the single-strand telomeric DNA binding domain of the Saccharomyces cerevisiae Cdc13 protein. Proc Natl Acad Sci U S A 2000; 97:6457-62; PMID:10841551; http://dx.doi.org/10.1073/pnas.97.12.6457 10841551 PMC18624

[50] Gardner RG, Nelson ZW, Gottschling DE. Degradation-mediated protein quality control in the nucleus. Cell 2005; 120:803-15; PMID:15797381; http://dx.doi.org/10.1016/j.cell.2005.01.016 15797381

[51] Polotnianka RM, Li J, Lustig AJ. The yeast Ku heterodimer is essential for protection of the telomere against nucleolytic and recombinational activities. Curr Biol 1998; 8:831-4; PMID:9663392; http://dx.doi.org/10.1016/S0960-9822(98)70325-2 9663392

[52] Foster SS, Zubko MK, Guillard S, Lydall D. MRX protects telomeric DNA at uncapped telomeres of budding yeast cdc13-1 mutants. DNA Repair (Amst) 2006; 5:840-51; PMID:16765654; http://dx.doi.org/10.1016/j.dnarep.2006.04.005 16765654

[53] Lydall D, Weinert T. Yeast checkpoint genes in DNA damage processing: implications for repair and arrest. Science 1995; 270:1488-91; PMID:7491494; http://dx.doi.org/10.1126/science.270.5241.1488 7491494

[54] Greenall A, Lei G, Swan DC, James K, Wang L, Peters H, Wipat A, Wilkinson DJ, Lydall D. A genome wide analysis of the response to uncapped telomeres in budding yeast reveals a novel role for the NAD+ biosynthetic gene BNA2 in chromosome end protection. Genome Biol 2008; 9:R146; PMID:18828915; http://dx.doi.org/10.1186/gb-2008-9-10-r146 18828915 PMC2760873

[55] Weinert TA, Kiser GL, Hartwell LH. Mitotic checkpoint genes in budding yeast and the dependence of mitosis on DNA replication and repair. Genes Dev 1994; 8:652-65; PMID:7926756; http://dx.doi.org/10.1101/gad.8.6.652 7926756

[56] Addinall SG, Holstein EM, Lawless C, Yu M, Chapman K, Banks AP, Ngo HP, Maringele L, Taschuk M, Young A, et al. Quantitative fitness analysis shows that NMD proteins and many other protein complexes suppress or enhance distinct telomere cap defects. PLoS genetics 2011; 7:e1001362; PMID:21490951; http://dx.doi.org/10.1371/journal.pgen.1001362 21490951 PMC3072368

[57] Holstein EM, Clark KR, Lydall D. Interplay between nonsense-mediated mRNA decay and DNA damage response pathways reveals that Stn1 and Ten1 are the key CST telomere-cap components. Cell Rep 2014; 7:1259-69; PMID:24835988; http://dx.doi.org/10.1016/j.celrep.2014.04.017 24835988 PMC4518466

[58] Weinert TA, Hartwell LH. The RAD9 gene controls the cell cycle response to DNA damage in Saccharomyces cerevisiae. Science 1988; 241:317-22; PMID:3291120; http://dx.doi.org/10.1126/science.3291120 3291120

[59] Zubko MK, Guillard S, Lydall D. Exo1 and Rad24 differentially regulate generation of ssDNA at telomeres of Saccharomyces cerevisiae cdc13-1 mutants. Genetics 2004; 168:103-15; PMID:15454530; http://dx.doi.org/10.1534/genetics.104.027904 15454530 PMC1448105

[60] Ngo GH, Balakrishnan L, Dubarry M, Campbell JL, Lydall D. The 9-1-1 checkpoint clamp stimulates DNA resection by Dna2-Sgs1 and Exo1. Nucleic Acids Res 2014; 42:10516-28; PMID:25122752; http://dx.doi.org/10.1093/nar/gku746 25122752 PMC4176354

[61] Ngo HP, Lydall D. Survival and growth of yeast without telomere capping by Cdc13 in the absence of Sgs1, Exo1, and Rad9. PLoS genetics 2010; 6:e1001072; PMID:20808892; http://dx.doi.org/10.1371/journal.pgen.1001072 20808892 PMC2924318

[62] Elfving N, Chereji RV, Bharatula V, Bjorklund S, Morozov AV, Broach JR. A dynamic interplay of nucleosome and Msn2 binding regulates kinetics of gene activation and repression following stress. Nucleic Acids Res 2014; 42:5468-82; PMID:24598258; http://dx.doi.org/10.1093/nar/gku176 24598258 PMC4027177

[63] Martinez-Pastor MT, Marchler G, Schuller C, Marchler-Bauer A, Ruis H, Estruch F. The Saccharomyces cerevisiae zinc finger proteins Msn2p and Msn4p are required for transcriptional induction through the stress response element (STRE). EMBO J 1996; 15:2227-35; PMID:8641288 8641288 PMC450147

[64] Sadeh A, Movshovich N, Volokh M, Gheber L, Aharoni A. Fine-tuning of the Msn2/4-mediated yeast stress responses as revealed by systematic deletion of Msn2/4 partners. Mol Biol Cell 2011; 22:3127-38; PMID:21757539; http://dx.doi.org/10.1091/mbc.E10-12-1007 21757539 PMC3164460

[65] Berry DB, Gasch AP. Stress-activated genomic expression changes serve a preparative role for impending stress in yeast. Mol Biol Cell 2008; 19:4580-7; PMID:18753408; http://dx.doi.org/10.1091/mbc.E07-07-0680 18753408 PMC2575158

[66] Jaspersen SL, Morgan DO. Cdc14 activates cdc15 to promote mitotic exit in budding yeast. Curr Biol 2000; 10:615-8; PMID:10837230; http://dx.doi.org/10.1016/S0960-9822(00)00491-7 10837230

[67] Bardin AJ, Boselli MG, Amon A. Mitotic exit regulation through distinct domains within the protein kinase Cdc15. Mol Cell Biol 2003; 23:5018-30; PMID:12832486; http://dx.doi.org/10.1128/MCB.23.14.5018-5030.2003 12832486 PMC162228

[68] Gilbert CS, Green CM, Lowndes NF. Budding yeast Rad9 is an ATP-dependent Rad53 activating machine. Mol Cell 2001; 8:129-36; PMID:11511366; http://dx.doi.org/10.1016/S1097-2765(01)00267-2 11511366

[69] Usui T, Ogawa H, Petrini JH. A DNA damage response pathway controlled by Tel1 and the Mre11 complex. Mol Cell 2001; 7:1255-66; PMID:11430828; http://dx.doi.org/10.1016/S1097-2765(01)00270-2 11430828

[70] Jia X, Weinert T, Lydall D. Mec1 and Rad53 inhibit formation of single-stranded DNA at telomeres of Saccharomyces cerevisiae cdc13-1 mutants. Genetics 2004; 166:753-64; PMID:15020465; http://dx.doi.org/10.1534/genetics.166.2.753 15020465 PMC1470748

[71] Sweeney FD, Yang F, Chi A, Shabanowitz J, Hunt DF, Durocher D. Saccharomyces cerevisiae Rad9 acts as a Mec1 adaptor to allow Rad53 activation. Curr Biol 2005; 15:1364-75; PMID:16085488; http://dx.doi.org/10.1016/j.cub.2005.06.063 16085488

[72] Lazzaro F, Sapountzi V, Granata M, Pellicioli A, Vaze M, Haber JE, Plevani P, Lydall D, Muzi-Falconi M. Histone methyltransferase Dot1 and Rad9 inhibit single-stranded DNA accumulation at DSBs and uncapped telomeres. EMBO J 2008; 27:1502-12; PMID:18418382 18418382 10.1038/emboj.2008.81 PMC2328446

[73] Ngo GH, Lydall D. The 9-1-1 checkpoint clamp coordinates resection at DNA double strand breaks. Nucleic Acids Res 2015; 43:5017-32; PMID:25925573; http://dx.doi.org/10.1093/nar/gkv409 25925573 PMC4446447

[74] Larrivee M, Wellinger RJ. Telomerase- and capping-independent yeast survivors with alternate telomere states. Nat Cell Biol 2006; 8:741-7; PMID:16767083; http://dx.doi.org/10.1038/ncb1429 16767083

[75] Costantini D, Monaghan P, Metcalfe NB. Prior hormetic priming is costly under environmental mismatch. Biol Lett 2014; 10:20131010; PMID:24522630; http://dx.doi.org/10.1098/rsbl.2013.1010 24522630 PMC3949371

[76] Rupec RA, Baeuerle PA. The genomic response of tumor cells to hypoxia and reoxygenation. Differential activation of transcription factors AP-1 and NF-kappa B. Eur J Biochem 1995; 234:632-40; PMID:8536713; http://dx.doi.org/10.1111/j.1432-1033.1995.632_b.x 8536713

[77] Laderoute KR, Webster KA. Hypoxia/reoxygenation stimulates Jun kinase activity through redox signaling in cardiac myocytes. Circ Res 1997; 80:336-44; PMID:9048653; http://dx.doi.org/10.1161/01.RES.80.3.336 9048653

[78] Seko Y, Takahashi N, Tobe K, Kadowaki T, Yazaki Y. Hypoxia and hypoxia/reoxygenation activate p65PAK, p38 mitogen-activated protein kinase (MAPK), and stress-activated protein kinase (SAPK) in cultured rat cardiac myocytes. Biochem Biophys Res Commun 1997; 239:840-4; PMID:9367856; http://dx.doi.org/10.1006/bbrc.1997.7570 9367856

[79] Abbott A. Researchers pin down risks of low-dose radiation. Nature 2015; 523:17-8; PMID:26135428; http://dx.doi.org/10.1038/523017a 26135428

[80] Leuraud K, Richardson DB, Cardis E, Daniels RD, Gillies M, O'Hagan JA, Hamra GB, Haylock R, Laurier D, Moissonnier M, et al. Ionising radiation and risk of death from leukaemia and lymphoma in radiation-monitored workers (INWORKS): an international cohort study. Lancet Haematol 2015; 2:e276-e81; PMID:26436129; http://dx.doi.org/10.1016/S2352-3026(15)00094-0 26436129 PMC4587986

[81] Calabrese E Hormesis: Once Marginalized, Evidence Now Supports Hormesis as the Most Fundamental Dose Response In: Mattson MP, Calabrese EJ, ed. Hormesis: A Revolution in Biology, Toxicology and Medicine. New York: Humana Press, 2010:15-56.

