# Supplementary material for: Costs, benefits and redundant mechanisms of adaption to chronic low-dose stress in yeast
Source: Cell Cycle. 2016 Aug 11;15(20):2732–41. doi: 10.1080/15384101.2016.1218104 (PMC5053569; doi:10.1080/15384101.2016.1218104)
Supplement: Supplemental Files [file kccy-15-20-1218104-s001.docx]

**Supplementary Table and Figures**

**Table S1 Strains used in this study.** All *S. cerevisiae* strains are in the W303 genetic

background *(ade2-1 can1-100 trp1-1 leu2-3,112 his3-11,15 ura3 GAL+ psi+ ssd1-d2*

*RAD5+).*


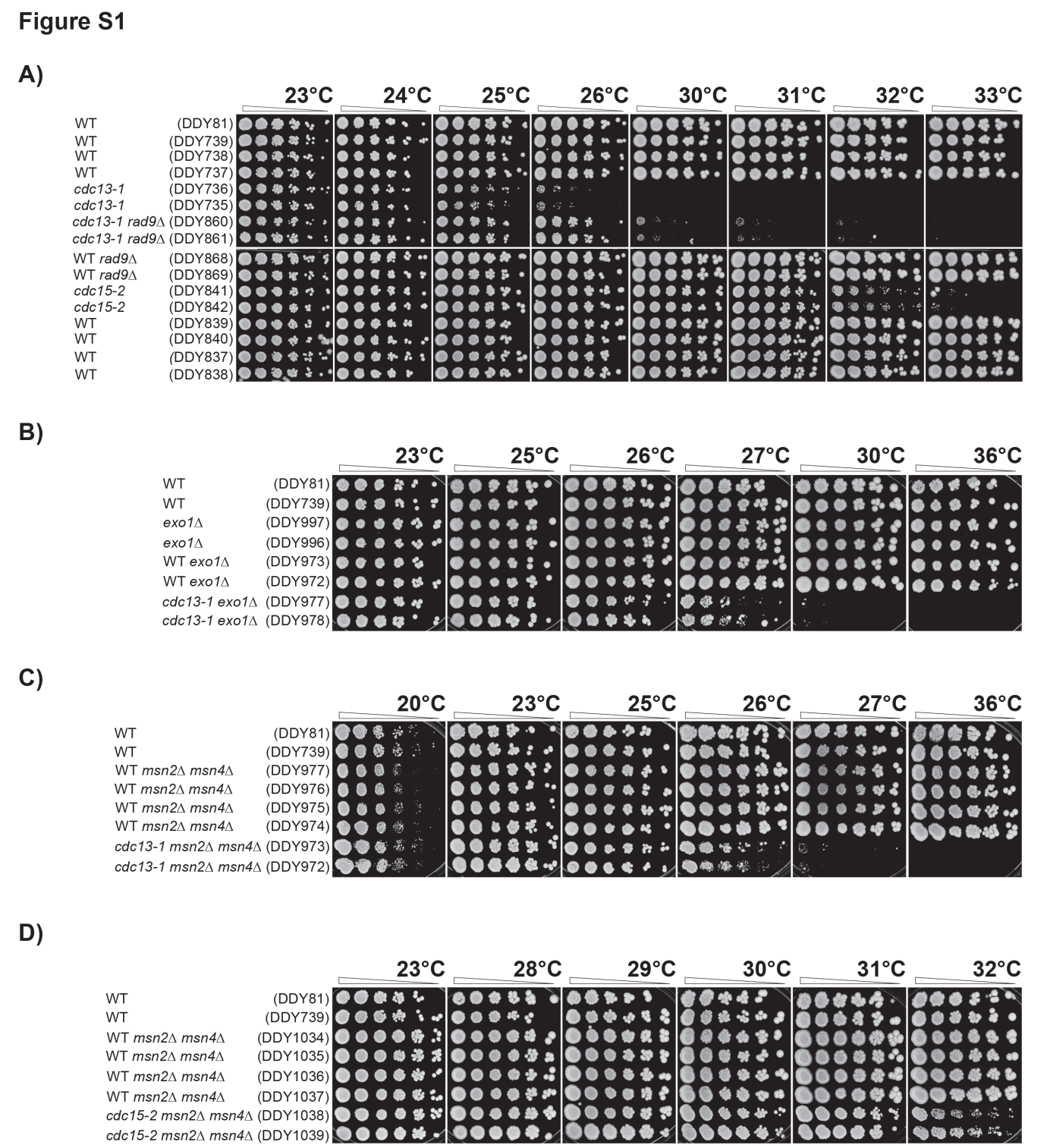


**Figure S1. The fitness of *cdc13-1* and *cdc15-2* mutants at a range of temperatures.**

Diploid strains with different genotypes were grown for one passage at 23°C on YEPD plates. Spot tests were performed as described in Figure 3. Photographs were taken after 72

hours. All strains shown at each temperature were grown on a single agar plate within A).

Strains’ details are shown in Supplementary Table 1.


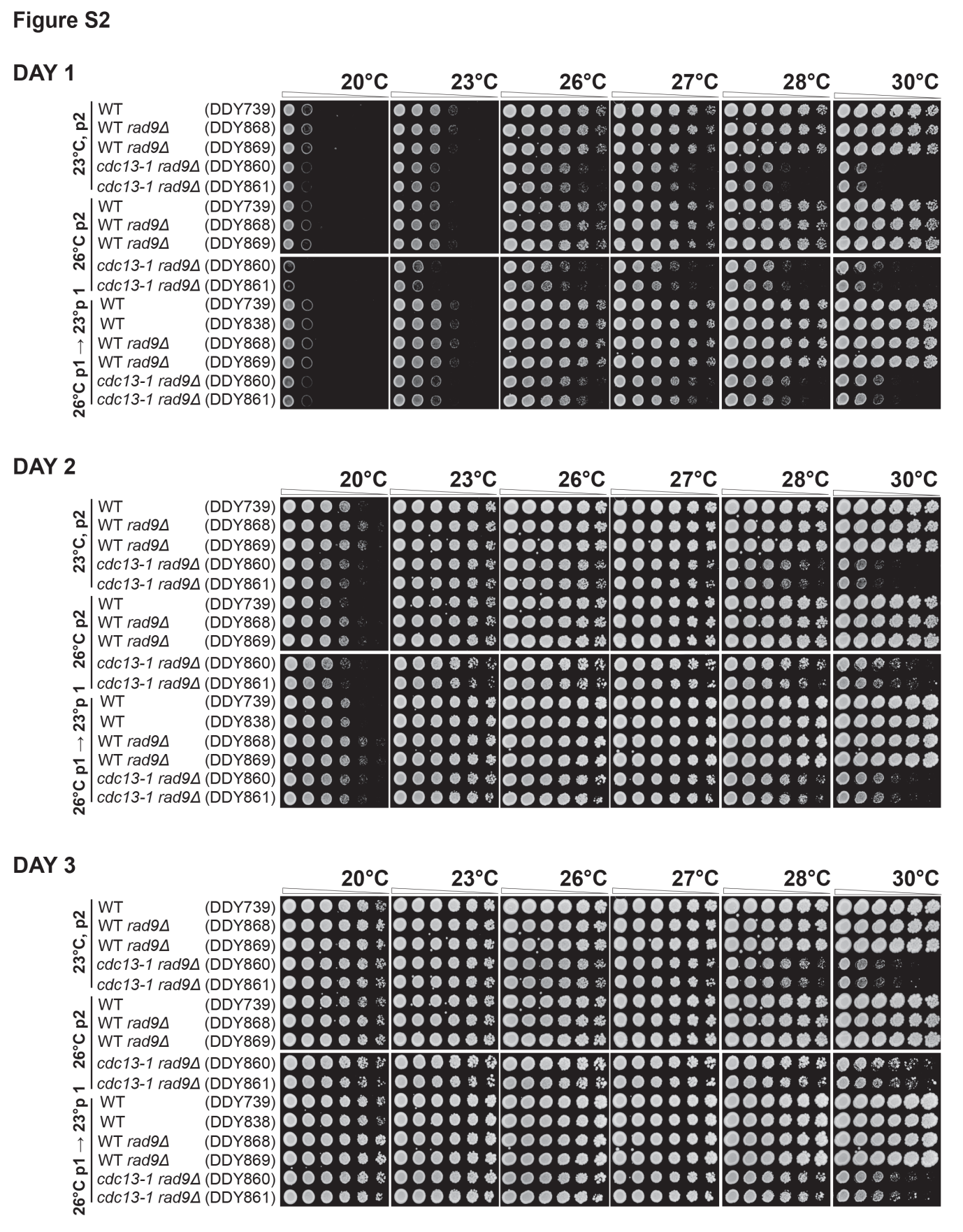


**Figure S2. The effects of adaptation to telomere stress in *cdc13-1 rad9∆* mutants.**

Pictures were taken periodically after 24, 48 and 72 hours of incubation. Experimental

design described in Figure 4.


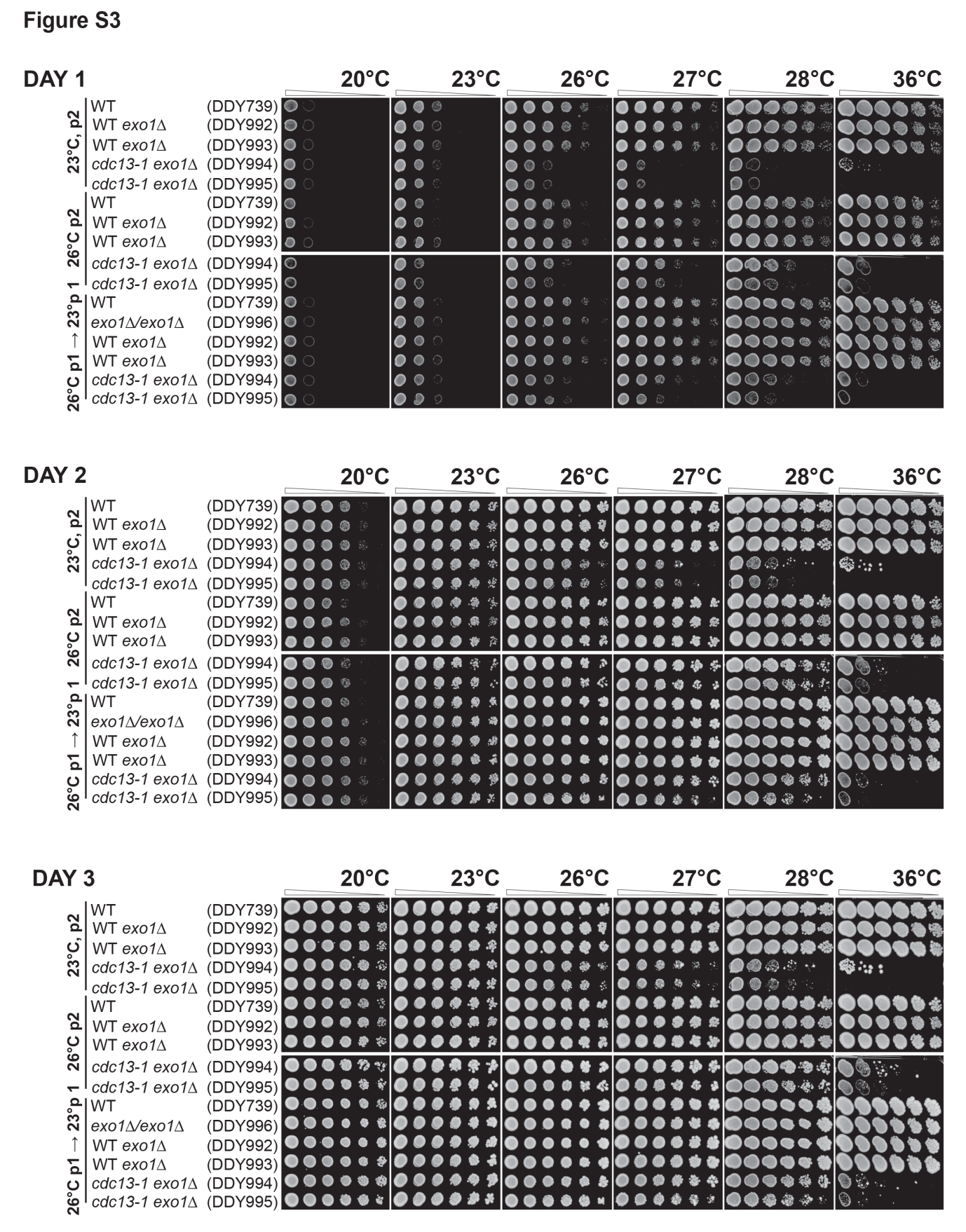


**Figure S3. The effects of adaptation to telomere stress in *cdc13-1 exo1∆* mutants.**

Pictures were taken periodically after 24, 48 and 72 hours of incubation. Experimental

design described in Figure 4.


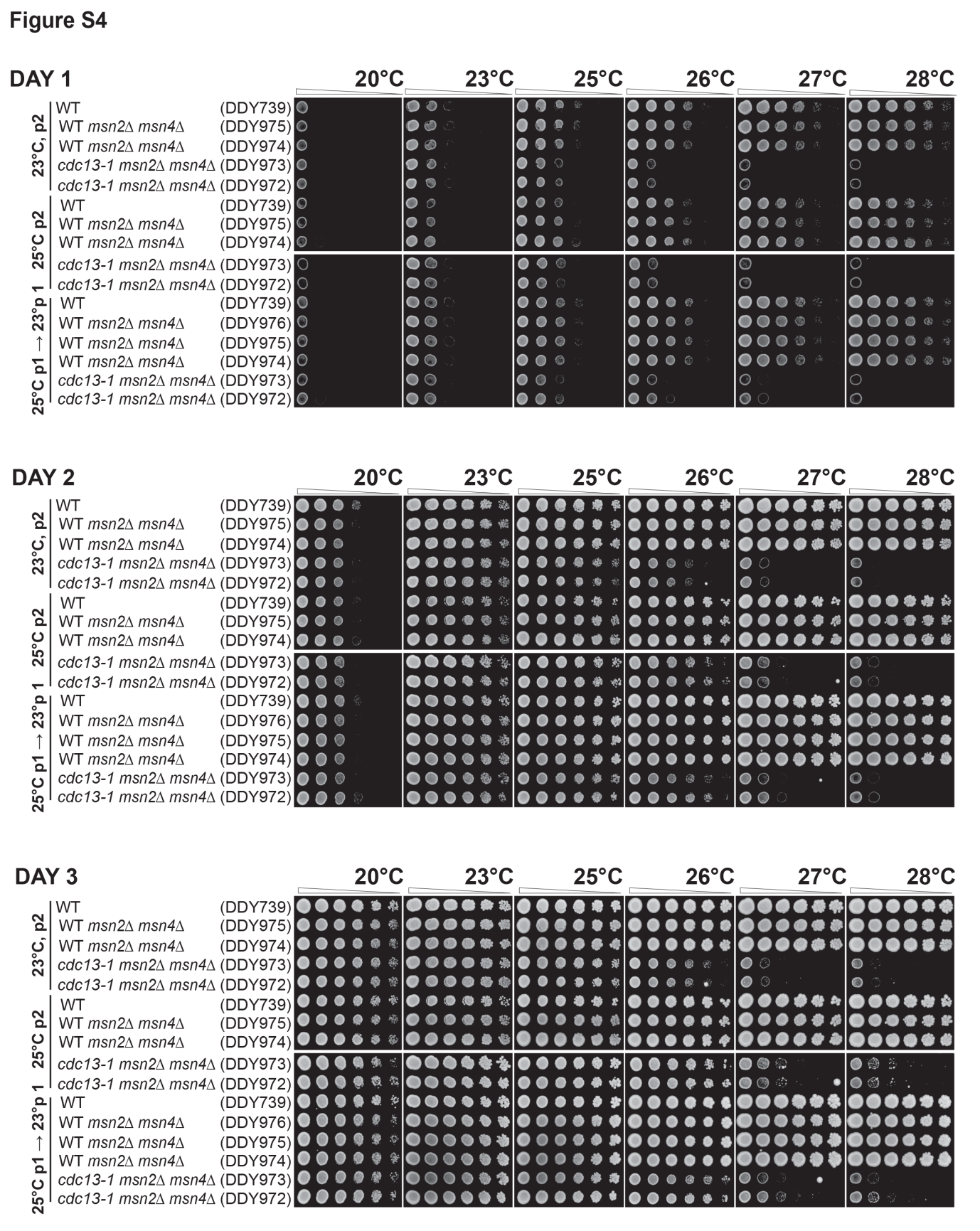


**Figure S4. The effects of adaptation to telomere stress in *cdc13-1 msn2∆ msn4∆***

**mutants.**

Pictures were taken periodically after 24, 48 and 72 hours of incubation. Experimental

design described in Figure 4.


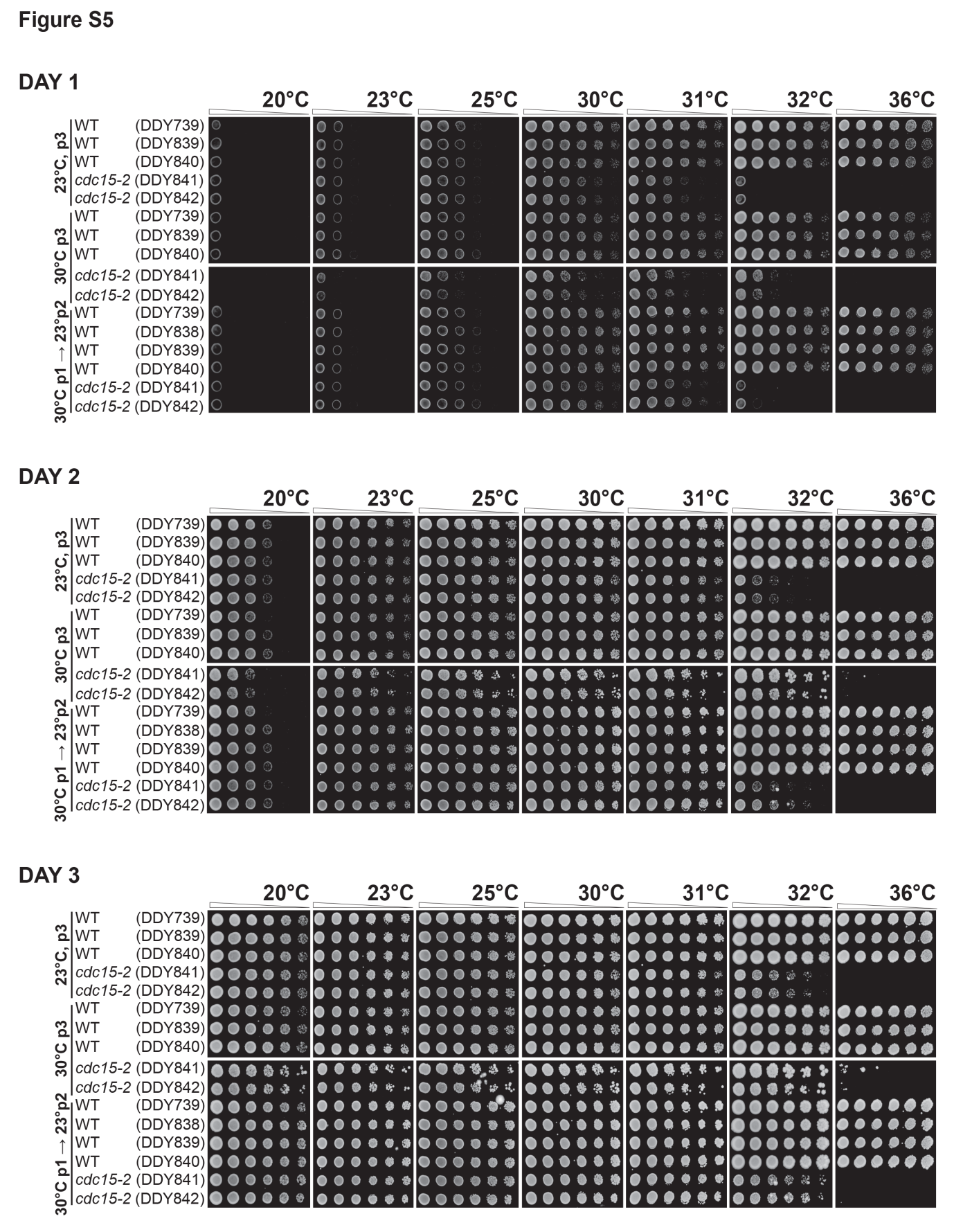


**Figure S5. The effects of adaptation to low level kinase inhibition in *cdc15-2* mutants.**

Pictures were taken periodically after 24, 48 and 72 hours of incubation. Experimental

design described in Figure 5.


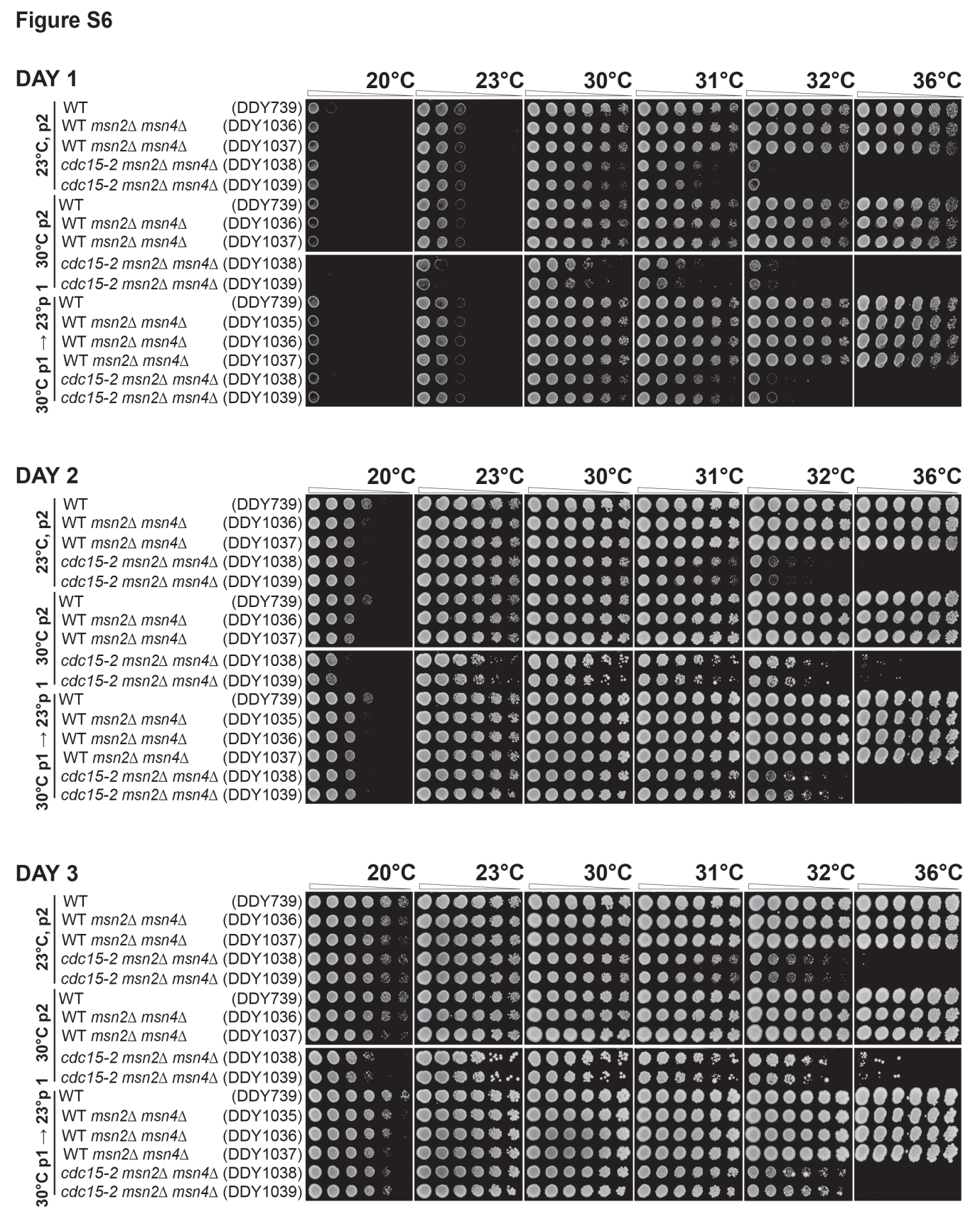


**Figure S6. The effects of adaptation to low level kinase inhibition in *cdc15-2 msn2∆***

***msn4∆* mutants.**

Pictures were taken periodically after 24, 48 and 72 hours of incubation. Experimental

design described in Figure 5.
